# Pathology and Therapeutics of Typhus Fever

**Published:** 1860-08

**Authors:** 


### SELECTIONS.

*Pathology and Therapeutics of Typhus Fever.*—The number of the Glasgow Medical Journal for January, 1860, contains an interesting paper on this subject by Dr. Jos. Bell, one of the physicians to the Glasgow Infirmary. The following are his concluding propositions:

1. That in numerous cases of typhus, about the fifth, sixth or seventh day of the attack, the impulse and systolic sound of the heart becomes feeble, and ultimately imperceptible.

2. That these symptoms indicate a morbid alteration in the structure of the muscular tissue of the heart, especially in the walls of the left ventricle.

3. That this alteration resembles the usual changes which result from congestion and inflammation of the muscular structure.

4. That the nature of this pathological change requires further examination and research, because the evidences on which the doctrine of its non-inflammatory origin rest, are not conclusive; the circumstances on which Louis and Stokes have placed reliance not being uniformly present.

5. That the beneficial influence of stimulants does not prove the non-inflammatory nature of the morbid change, because in asthenic inflammation a stimulating treatment is always necessary.

6. That whether or not the pathological alteration be owing to inflammation, the softening must be regarded as one of the secondary effects of typhus.

7. That the proper treatment is to maintain the action of the heart by stimulants.

8. That in cases of cerebral and pulmonary disturbance arising in connection with cardiac softening, a stimulating plan of treatment is indicated.

9. That the presence or absence of the physical symptoms diagnostic of softened heart, may be relied on as affording trustworthy evidence by which the asthenic nature of these cerebral and pulmonary affections can be determined.

From these propositions it follows as a *carollary*, that it is the duty of the physician to devote the strictest attention to the action of the heart, especially as regards its impulse and sounds, throughout the course every case of typhus.—*Amer. Jour. Med. Sci.*

